# Spatiotemporal Cofilin Signaling, Microglial Activation, Neuroinflammation, and Cognitive Impairment Following Hemorrhagic Brain Injury

**DOI:** 10.3390/cells12081153

**Published:** 2023-04-13

**Authors:** Daniyah A. Almarghalani, Xiaojin Sha, Robert E. Mrak, Zahoor A. Shah

**Affiliations:** 1 Department of Pharmacology and Experimental Therapeutics, College of Pharmacy and Pharmaceutical Sciences, University of Toledo, Toledo, OH 43614, USA; 2 Department of Pathology, College of Medicine, The University of Toledo, Toledo, OH 43614, USA; 3 Department of Laboratory Medicine and Pathology, University of Washington, Seattle, WA 98195, USA; 4 Department of Medicinal and Biological Chemistry, University of Toledo, Toledo, OH 43614, USA

**Keywords:** cofilin, ICH, microglial activation, neuroinflammation, post-stroke cognitive impairment, cofilin rods/aggregates

## Abstract

Intracerebral hemorrhage (ICH) is a significant health concern associated with high mortality. Cofilin plays a crucial role in stress conditions, but its signaling following ICH in a longitudinal study is yet to be ascertained. In the present study, we examined the cofilin expression in human ICH autopsy brains. Then, the spatiotemporal cofilin signaling, microglia activation, and neurobehavioral outcomes were investigated in a mouse model of ICH. Human autopsy brain sections from ICH patients showed increased intracellular cofilin localization within microglia in the perihematomal area, possibly associated with microglial activation and morphological changes. Various cohorts of mice were subjected to intrastriatal collagenase injection and sacrificed at time points of 1, 3, 7, 14, 21, and 28 days. Mice suffered from severe neurobehavioral deficits after ICH, lasting for 7 days, followed by a gradual improvement. Mice suffered post-stroke cognitive impairment (PSCI) both acutely and in the chronic phase. Hematoma volume increased from day 1 to 3, whereas ventricle size increased from day 21 to 28. Cofilin protein expression increased in the ipsilateral striatum on days 1 and 3 and then decreased from days 7 to 28. An increase in activated microglia was observed around the hematoma on days 1 to 7, followed by a gradual reduction up to day 28. Around the hematoma, activated microglia showed morphological changes from ramified to amoeboid. mRNA levels of inflammatory [tumor necrosis factor-α (TNF-α), interleukin 1β (IL-1β), and interleukin-6 (IL-6) and anti-inflammatory markers [interleukin-10 (IL-10), transforming growth factor-β TGF-β, and arginase I (Arg1)] increased during the acute phase and decreased in the chronic phase. Blood cofilin levels increased on day 3 and matched the increase in chemokine levels. slingshot protein phosphatase 1 (SSH1) protein, which activates cofilin, was increased from day 1 to 7. These results suggest that microglial activation might be the sequel of cofilin overactivation following ICH, leading to widespread neuroinflammation and consequent PSCI.

## 1. Introduction

The global prevalence of intracerebral hemorrhage (ICH) was 17.9 million, accounting for 3 million deaths in 2017 [1]. Fifty percent of ICH patients die within the first month, 50% become severely disabled, and surgical intervention is the only treatment option [2,3,4]. Primary injury develops in the first few hours due to hematoma expansion that increases intracranial pressure, leading to herniation and death. Secondary injury is attributed mainly to hematoma expansion and oozing out of blood products in the hematoma. Degradation products of the hematoma, such as hemin, thrombin, and ferrous iron, initiate glial activation, neuronal damage, neuroinflammation, and reactive oxygen species formation, thus leading to neurological impairments [3,5]. Microglia, the primary immune cells in the central nervous system, account for 10–15% of all cells in the brain [6,7,8,9,10], are activated within minutes of brain injury, and persist for a month [11,12]. Different phenotypes of microglia contribute to secondary brain injury and neuroinflammation. The two main microglial phenotypes, M1 and M2, reflect contrasting effects on the inflammatory response [13]; the M1 phenotype enhances inflammation and releases inflammatory mediators, such as tumor necrosis factor-α (TNF-α), Interleukin 1β (IL-1β), and interleukin-6 (IL-6), whereas the M2 phenotype inhibits inflammatory responses and is responsible for debris removal and tissue repair. Activated microglia and persistent neuroinflammation contribute to cognitive impairment in such conditions as Parkinson’s disease [14], aging, Alzheimer’s disease [15], ischemic stroke [16,17], hemorrhagic stroke [18], and some psychiatric disorders [19].

Cofilin is an actin-depolymerizing protein that plays a crucial role in the actin filament turnover [20]. Cofilin regulates actin dynamics by maintaining globular actin (G-actin) monomers through depolarizing and severing filamentous actin (F-actin) [20,21]. Multiple kinases and phosphatase processes modulate cofilin phosphorylation/dephosphorylation. Cofilin is inactivated by LIM and TES kinases through phosphorylation at serine-3 residue (Ser3) but is reactivated by slingshots (SSH), chronophin, and phosphoprotein phosphatases (PP1/PP2A) [22,23,24,25]. Persistent cofilin activation results in saturated actin filaments and the formation of cofilin–actin rods, which are involved in neurotoxicity, impaired synaptic integrity, and dendritic spine loss [26]. The three major mechanisms for cofilin rods/aggregates include cofilin dephosphorylation, high cofilin/F-actin ratio, and cofilin oxidation [27]. In addition, cofilin is implicated in cell death as activated cofilin translocates to mitochondria and increases mitochondrial permeability, releasing cytochrome C and activating caspases [28,29]. A recent study on four ischemic mouse models showed that cofilin activation leads to cofilin–actin rod formation in neuronal processes [30]. Our previous in vitro studies have demonstrated that cofilin knockdown restores the cell viability [31] and decreases the microglia activation [31,32]. Treatment with a newly synthesized novel cofilin inhibitor (CI) improved neuronal viability and diminished microglial activation and neuroinflammation in an in vitro ICH model [33]. We have also demonstrated that cofilin knockdown by siRNA before ICH reduces neuronal apoptosis, microglial activation, and oxidative stress and improves neurobehavioral deficits [34]. 

This study investigated cofilin expression, microglial activation, and its morphological alteration in the human ICH brain autopsy specimens. Next, we looked at spatiotemporal cofilin signaling, microglial activation, and neuroinflammation in mice ICH model, which was complemented by the assessment of infarct volume, ventricle size, motor dysfunctions and post-stroke cognitive impairment (PSCI). Additionally, we also measured plasma cofilin and cytokine levels.

## 2. Materials and Methods

### 2.1. Human Autopsy Brain Sections

For immunohistochemistry, autopsy paraffin-embedded brain sections were obtained from tissue archives of the University of Toledo, Department of Pathology. The study included 8 control cases and 10 hemorrhagic brain injury cases (Table S1). An experienced pathologist selected tissue sections adjacent to intracerebral hemorrhage from the study subjects and corresponding sections from the control subjects with no history of brain injury. The post-mortem delay ranged from 24–72 h for controls and 15–68 h for patients with hemorrhagic brain injury. The IBC protocol was reviewed and approved by the IBC committee to work on human autopsy brain sections. The current study does not include human subjects, radioactive substances, or pathogens. All animal work was reviewed and approved by the University of Toledo’s Institutional Animal Care and Use Committee (IACUC). The animal use procedures complied with University guidelines, State and Federal regulations, and the standards of the “Guide for the Care and Use of Laboratory Animals”.

### 2.2. Animals 

All animal studies were conducted according to the National Institutes of Health guidelines and were approved by the University of Toledo Animal Care and Use Committee. 

### 2.3. Experimental Design 

ICH was induced by injecting collagenase into the left striatum of mice using stereotaxic injections (Stoelting, Wood Dale, IL, USA). Under isoflurane anesthesia, a small hole in the skull above the left striatum of each mouse was made, and a Hamilton syringe needle was inserted to deliver the collagenase (0.6 µL, 0.09 units). The stereotaxic coordinates used for collagenase injection were 1 mm anterior to bregma, 2 mm lateral to the midline, and 3 mm depth from the skull surface. The needle was slowly withdrawn (after 10 min), and the wound was sutured. Mice were placed on a heated pad to recover from the surgery and then transferred to a clean cage. Sham mice received only insertion of the needle without collagenase injection.

For the spatiotemporal signaling of cofilin, a total of 126 C57BL/6 male mice (8–14-week-old) were obtained from Jackson Laboratory and randomly divided into the control group (sham surgery) and the ICH group. Animals were assessed for neurobehavioral parameters [such as, rotarod, grip strength, and neurologic deficit scores (NDS) performed by an expert investigator blinded to the study plan, and findings were recorded for all animals and compared among ICH groups 24 h before the hemorrhagic injury and after 1, 3, 7, 14, 21, and 28 days. T-maze was used to evaluate spatial working memory in the acute and chronic periods. Animals were sacrificed at different times, and brain and blood samples were collected to perform various biological assays. Different cohorts of animals were sacrificed at 1, 3, 7, 14, 21, and 28 days (acute phase 1 to 3 days and chronic phase 7 to 28 days). All efforts were made to minimize the number of animals used and their suffering. We observed no deaths in this study.

### 2.4. Neurobehavioral Tests

A description of all behavioral tests is provided in detail in the Supplementary Materials.

### 2.5. Western Blotting

For Western blotting (WB) analysis, different cohorts of mice were euthanized with CO_2_, and brains were removed and collected to examine potential changes in the expression levels of cofilin at different time points (days 1, 3, 7, 14, 21, and 28). Brain tissues were collected from the hemorrhagic sides (ipsilateral) and non-hemorrhagic sides (contralateral) using a 1 mm-diameter micro-punch needle. Brain tissues were homogenized in a buffer containing 10 mM HEPES (pH 7.9), 1.5 mM MgCl_2_, 10 mM KCl, 10 mM DTT, 4 mM PMSF, 10% NP40, 100 mM Na OrthoV, 1 M NaF, 200 mM NaPP, and protease inhibitor cocktail (Sigma–Aldrich, St. Louis, MO, USA). Protein concentrations were measured using Bradford reagent following the manufacturer’s instructions (Bio-Rad, Hercules, CA, USA). The samples were analyzed by loading equivalent amounts of protein onto 12% SDS-polyacrylamide gels. Proteins were separated using gel electrophoresis, transferred onto a polyvinylidene fluoride membrane (PVDF), and blocked with 5% BSA for 1 h to inhibit non-specific binding. The membranes were incubated overnight at 4 °C with the following primary antibodies: rabbit anti-cofilin (1:1000; Cell Signaling Technology, Inc. Denvers MA, USA), rabbit anti-p-cofilin (1:500; cell signaling), rabbit anti-ROCK1 (1:1000; Cell Signaling), rabbit anti-SSH1 (1:1000; Abcam, Waltham, MA, USA), rabbit anti-LIMK1 (1:1000; Cell Signaling), rabbit anti-p-LIMK (1:1000; Cell Signaling), mouse anti-synaptophysin (1:1000; Abcam), and mouse anti-PSD95 (1:1000, Cell Signaling). The next day, the blots were washed with TBST and incubated with horseradish peroxidase (HRP)-conjugated secondary antibodies (goat anti-rabbit and goat anti-mouse, Cell Signaling) for 1 h at room temperature. The images were captured using a Syngene Imaging System (Frederick, MD, USA). The images were analyzed using ImageJ Software, Version 1.53t 24. GAPDH (1:1000; Cell Signaling Technology) was used as a loading control. 

### 2.6. Histology

For histological assessments, sham and ICH mice were anesthetized with Ketamine/Xylazine (100 mg/kg and 10 mg/kg, respectively, i.p.) and then transcardially perfused with 1X PBS, followed by 4% paraformaldehyde. Mice brains were isolated and placed in 4% paraformaldehyde for 24 h, followed by paraffin-embedded sections (5 µm). Brain sections were deparaffinized, followed by Luxol fast blue/cresyl violet staining to measure brain ventricles and hematoma volume. Hematoxylin and eosin (H&E) staining was used to examine brain lesions. Additional sections were used for immunohistochemistry.

### 2.7. Immunohistochemistry

Brain sections from human subjects and different cohorts of mice were deparaffinized, followed by antigen retrieval in a pressure cooker for 15 min, washed with 1XPBS for 5 min, and finally blocked with 5% BSA in TBST for 2 h on a rocker. The primary antibodies were added and incubated overnight at 4 °C, 1:1000, rabbit anti-IBA1 (Wako, Japan) and 1:1000, mouse anti-cofilin (Abcam, Waltham, MA, USA) and mouse anti-F-actin (Thermofisher Scientific, Waltham, MA, USA). The next day, slides were washed with 1XPBS, and the secondary antibodies were added and incubated for 1 h at room temperature (Taxes red labeled donkey anti-rabbit IgG (1:1000; Jackson, Immunoresearch, West Grove, PA, USA) and Alexa Fluor donkey anti-mouse IgG (1:1000; Jackson, Immunoresearch). Slides were washed with 1X PBS and mounted with DAPI (Molecular Probes, Eugene, OR, USA). The images used to assess the localization of cofilin in microglia and cofilin with F-actin were detected using fluorescent microscopy. Cofilin and microglia intensity around the hemorrhage was determined using Image J software (NIH, Bethesda, Maryland, USA), and quantitative analysis of microglia was assessed by counting the activated cells around the hematoma using ImageJ software (NIH).

### 2.8. Microglia Morphology Analysis

Morphological changes of microglia in the human and mouse brain sections were quantified using ImageJ and appropriate plugins of FracLac for the ImageJ analysis [35]. Images were converted to photomicrographs of binary and outline images. We used a box-counting dimension method that is exquisitely sensitive to the morphological features of microglia. We quantified cell complexity, shape, size, and length using fractal dimensions, span ratio, density, and radius ratio [36,37,38]. The analysis was applied to single-cell microglia outline images. 

### 2.9. Quantitative Real Time-PCR

Gene expression was performed by quantitative real-time PCR (qRT-PCR). Total RNA from the ipsilateral and contralateral regions of different time points of ICH and sham brain tissues was isolated using TRIzol reagent (Invitrogen, Carlsbad, CA, USA). The complementary DNA (cDNA) was synthesized using the iScript cDNA synthesis kit (Bio-rad, Hercules, CA, USA). The mRNA expression level of the brain tissues was detected by qRT-PCR via SsoAdvanced Universal SYBR Green Supermix (Bio-Rad) using an Applied Biosystems (Waltham, MA, USA) Real-Time-PCR instrument system. The 18S gene was used as a housekeeping control. Gene abundance was analyzed using the ^2−ΔΔ^CT method to calculate relative changes in the expression of the target gene relative to the control. Results were shown as fold changes relative to the control group. All the sequences of the primers are listed in Table S2 (Supplementary Materials). A total of 28 mice were used in this experiment.

### 2.10. ELISA

Plasma cofilin was measured using a commercially available mouse cofilin enzyme-linked immunosorbent assay (ELISA) kit (Biotechne, Minneapolis, MN, USA). Briefly, 10 μL of each plasma sample mixed with 90 μL diluent was applied to the mouse cofilin kit to estimate cofilin concentrations (pg/mL) according to the manufacturer’s instructions.

### 2.11. Cytokine Proteome Profiler Array 

The inflammatory cytokine/chemokine levels in the serum of sham mice and ICH mice on day 3 after ICH were analyzed using the Mouse Cytokine Proteome Profiler Array Panel A kit (R&D system, Minneapolis, MN, USA) according to the manufacturer’s instructions. Briefly, 200 µL serum samples mixed with array buffers and detection antibody cocktails were incubated overnight at 4 °C on a platform shaker with pre-coated captured antibody array membranes. The signal was developed with West-Femto Maximum Sensitivity Substrate (Thermofisher Scientific), and images were captured with a Syngene Imaging System (Frederick, MD, USA). ImageJ-protein array was used to quantify spot density; then, the background signal was subtracted from each value. Finally, we compared the spot density on different arrays to evaluate the relative changes in cytokine/chemokine levels between sham and day 3 ICH groups. A total of 8 mice were used in this experiment (*n* = 4 sham and *n* = 4 ICH).

### 2.12. Statistics and Data Quantification

Data are shown to be normally distributed, which is confirmed by Shapiro–Wilk’s test. Quantifiable data were reported as mean ± SEM and statistical significance was set at *p* < 0.05. One or two-way ANOVA, followed by Sidak post hoc comparisons, was used to compare data for more than two groups. In addition, repeated-measures ANOVA followed by Sidak post hoc comparisons was used for neurobehavioral data analysis when equal variances and normal distribution were observed. Two-tailed unpaired Student’s *t*-test was used to compare the mean of the two groups. All statistics were performed using GraphPad Prism version 9.2 (GraphPad Software, San Diego, CA, USA).

## 3. Results

### 3.1. Cofilin Expression Is Increased in Human ICH Autopsy Brain Sections

**Figure 1:**
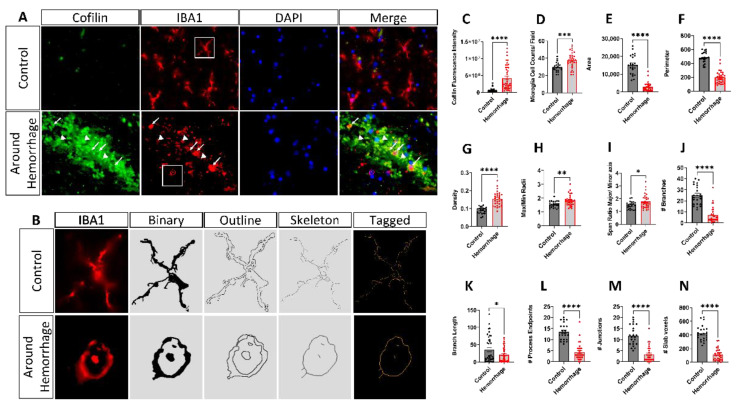
Cofilin and microglia are activated in human ICH brain sections. Human autopsy ICH and control brain sections were assessed for cofilin and microglial activation using immunofluorescence. (**A**) A significant increase is observed in cofilin and microglial activation around hemorrhagic areas after ICH compared to the control group (scale bar 20 µm; white arrows represent localization of cofilin in microglia, and arrowheads represent the cofilin activation in the neuropil area). (**B**) Analysis of microglia morphologies: binary images are outlined and then skeletonized to study the morphological alteration of microglia in the ICH and control groups using ImageJ plugin protocols. (**C**,**D**) Quantification analysis of cofilin intensity and microglia counts were performed using ImageJ software. (**E**–**N**) Summary data and statistical analysis of microglial morphology (shape, complexity, branch, and process) using FracLac protocols. Data are expressed as mean ± SEM, where *p* < 0.05 was considered significant (*n* = 8 control; *n* = 10 ICH; unpaired Student’s *t*-test). Anti-cofilin (a marker of active cofilin) and anti-IBA1 (a marker of active microglia/macrophage). * *p* < 0.05; ** *p* < 0.01; *** *p* < 0.001; **** *p* < 0.0001).

To evaluate cofilin expression and microglial activation in human ICH autopsy brain sections, we performed immunohistochemistry using cofilin (mouse monoclonal) as a cofilin marker and IBA1 (ionized Ca^2+^—binding adapter molecule 1, rabbit monoclonal) as an activated microglial marker. We found that cofilin rods/aggregates were highly expressed around the hematoma in ICH subjects (Figure 1). Cofilin intensity was significantly higher in ICH compared to the controls (*t*-test; *p* < 0.0001) (Figure 1A,C). We observed significant differences in microglia counts/field between ICH and controls (*t*-test; *p* < 0.0004) (Figure 1B,D). Intracellular cofilin rods/aggregates were localized to activated microglia in the ICH group and associated with microglia morphology changes compared to the control group (Figure 1). Male and female ICH patients showed increased cofilin intensity and microglia count around the hemorrhage compared to control groups (Figure S1). Some patients in the ICH group also showed cofilin rods/aggregates around the nucleus, raising the question of the impact of nuclear cofilin rods on transcription (Figure S2). We next characterized microglial morphological alterations in ICH and control brain sections using FracLac for ImageJ analysis. We observed activated amoeboid microglia around the hematoma area in the ICH group compared to the ramified to de-ramified microglia in the control group. We measured microglial complexity (area and perimeter), span ratio, density, radius length, and branches (processes) features using ImageJ and observed microglial area and perimeter dramatically decreased, but the thickness, span ratio, and max/min radius length were increased significantly in the ICH group compared to the control group (Figure 1E–I). The number of microglia branches, process endpoints, junctions, and slab voxels, as well as the length of the branches, were markedly decreased in the ICH group compared to the control group, indicating that amoeboid microglia were the dominant form following ICH (Figure 1J–N). These results suggest that cofilin and microglia are activated and upregulated around hematomas in the ICH group contributing to secondary injury.

### 3.2. Changes in Neurofunctional Parameters after ICH

**Figure 2:**
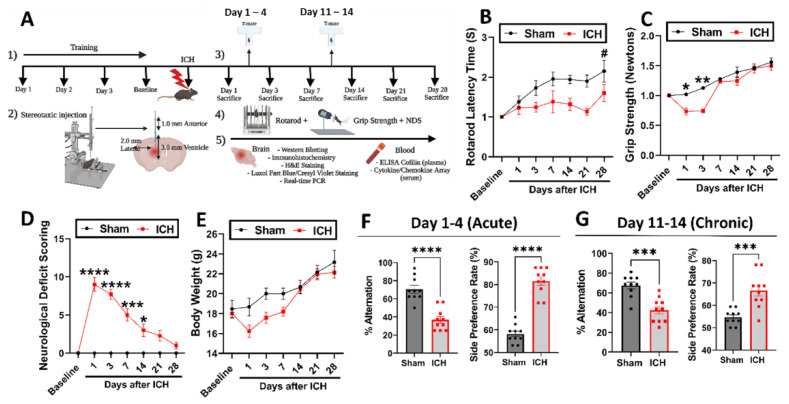
Experimental design and assessment of motor and cognitive deficits following ICH. Male mice were subjected to ICH and assessed for motor and cognitive deficits at different time points (**A**). Schematic of experimental design. Animals were assigned randomly to seven groups, sham, and six ICH groups, with different time points (1, 3, 7, 14, 21, and 28 days). Different cohorts of mice were subjected to intra-striatal collagenase injection-induced ICH, and sham mice had an insertion of only the needle (total of 126; *n* = 28 control (sham) and *n* = 98 (ICH). (**B**) The reduction in rotarod latency time following ICH was significantly improved by day 28. (**C**) Following ICH, mice exhibited a sustained functional deficit of forelimb strength that lasted for 7 days, with a significant improvement observed on days 21 and 28. (**D**) NDS was observed on day 1 and lasted until day 14, followed by a significant decrease on day 21. (**E**) The mice exhibited a decrease in body weight after ICH, and recovery was detected on day 7 and progressively increased on day 28. (**F**,**G**) The spontaneous alternation rate was significantly reduced in ICH compared to the sham group on days (1–4) and (11–14), whereas the side preference rate increased dramatically in the ICH group compared to the sham group. Repeated measure one-way ANOVA or two-way ANOVA followed by Sidak’s post hoc comparisons for rotarod, grip strength, NDS, and body weight; and unpaired Student’s *t*-test for T-maze was used for analysis. Data are expressed as mean ± SEM, where *p* < 0.05 was considered significant. * Difference within groups relative to baseline. (*n* = 7 mice for rotarod; grip strength; NDS and body weight, and *n* = 10 mice per group for T-maze test; ns = not significant; * *p* < 0.05; ** *p* < 0.01; *** *p* < 0.001; **** *p* < 0.0001). # Difference of contralateral and ipsilateral regions in different time-points after ICH (# *p* < 0.05).

A schematic of the experimental design is presented in Figure 2A. Neurobehavioral parameters (rotarod, grip strength, and NDS) and body weight measurements were assessed before (baseline) and after ICH at various time points (1, 3, 7, 14, 21, and 28) and compared with the sham group. The reduction in the rotarod latency time following ICH was not significant on days 1, 3, 7, 14, and 21, and a significantly enhanced latency time was observed on day 28, suggesting motor learning during repeated testing (*p* < 0.01; Figure 2B). Following ICH, mice exhibited a sustained functional deficit of the forelimb strength lasting up to day 7, and a significant improvement was observed on days 21 and 28 (*p* < 0.001; Figure 2C). NDS observed on day 1 lasted until day 14 day, followed by a significant decrease on day 21, indicating a recovery phase following ICH (Figure 2D). The mice exhibited a reduction in body weight after ICH, and the recovery was noted from days 14 to 28 (Figure 2E). 

We evaluated acute and chronic PSCI after ICH using T-maze for spontaneous alternation and side preference on days 1, 2, 3, and 4 (acute) and days 11, 12, 13, and 14 (chronic) phases. We found a significant reduction in the percent alternation rate in the ICH (acute (*p* < 0.0001) and chronic (*p* < 0.001) phases) compared to the sham group (Figure 2F,G). The percent of side preference was also significantly increased in the ICH group compared to the sham in acute (*p* < 0.0001) and chronic (*p* < 0.0001) phases, indicating impaired spatial alternation following ICH (Figure 2F,G). 

### 3.3. Spatiotemporal Cofilin Signaling in Mice after ICH

**Figure 3:**
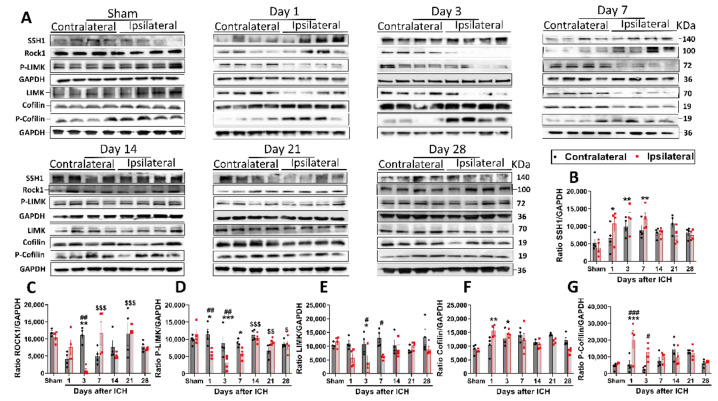
Cofilin and its upstream/downstream signaling. Animals from different cohorts were sacrificed on days 1, 3, 7, 14, 21, and 28, brains dissected, and contralateral and ipsilateral regions isolated for Western blotting. (**A**) Representative blots from different cohorts with different antibodies. A total of 25 µg of protein was loaded into each gel lane. The sham group was used as a control, GAPDH as a loading control, SSH1, ROCK1, *p*-LIMK, and LIMK as markers for cofilin signaling; cofilin was used as a marker of active cofilin, and p-cofilin was used as a marker of inactive cofilin. (**B**–**G**) Quantifying band intensities (normalized to GAPDH, *n* = 4 mice per group). (**B**) SSH1 was significantly increased on days 1, 3, and 7 after ICH compared to the sham group on the ipsilateral side, while a decrease was observed up to day 28. (**C**) ROCK1 was significantly decreased on day 3; (**D**) p-LIMK was significantly reduced on days 1, 3, and 7, and (**E**) LIMK1 was markedly reduced on days 3 and 7 (**B**–**E**). No significant changes in SSH1, ROCK1, p-LIMK, and LIMK1 were observed on the contralateral side. (**F**) Cofilin was significantly overexpressed on day 3 compared with the sham group, and a decrease was observed from day 3 up to day 28. In the ipsilateral region (around the hemorrhage), cofilin was significantly increased on days 1 and 3 compared with the sham group. Cofilin expression was decreased from day 7 to 28 compared with the sham group. (**G**) p-cofilin was significantly increased on day 1 and decreased from day 3 to 28 compared to the sham group. No significant changes were noted on the contralateral side. Two-way ANOVA followed by Sidak’s post hoc comparisons was used for analysis. Data are expressed as mean ± SEM, where *p* < 0.05 was considered significant. * Difference within groups relative to the sham group (* *p* < 0.05; ** *p* < 0.01; *** *p* < 0.001). $ Difference within ICH groups vs. ICH on day 3 in the ipsilateral region ($ *p* < 0.05; $$ *p* < 0.01; $$$ *p* < 0.001) and # Difference of contralateral and ipsilateral regions in different time-points after ICH (# *p* < 0.05; ## *p* < 0.01; ### *p* < 0.001).

Mouse brains were dissected, and WB was performed to observe cofilin expression levels in hemorrhagic (ipsilateral) and (non-hemorrhagic (contralateral) hemispheres. To understand cofilin-related signaling, we investigated upstream and downstream signaling cascades (Figure 3B–E). The expression of SSH1 was significantly increased on days 1, 3, and 7 after ICH (Figure 3B), suggesting cofilin overactivation. We observed significant downregulation of ROCK1 on day 3 (Figure 3C), p-LIMK1 on days 1, 3, and 7 (Figure 3D), and LIMK1 on day 3 (Figure 3E) on the hemorrhagic side after ICH compared with the sham group. However, no significant changes were observed in SSH1, ROCK1, p-LIMK, and LIMK1 on the contralateral side from day 1 to day 28 (Figure 3B–E). Cofilin expression was significantly increased in the ipsilateral area on days 1 and 3 and decreased from day 7 to day 28 compared to the sham group (*p* < 0.01, Figure 3F). Cofilin expression increased on days 3 and 7, and a decrease was observed from day 7 through day 28 in the contralateral region (Figure 3F), suggesting cofilin offside effects on the contralateral hemisphere. The expression of p-cofilin was significantly increased on day 1 and decreased from day 3 until day 28 in the ipsilateral region compared to the sham group. No significant changes were observed on the contralateral side (Figure 3G). These observations suggest that cofilin overactivation at different time points after ICH could be mediated by the increased SSH1/LIMK1 activity.

### 3.4. Synaptic Dysfunction in Mice after ICH

**Figure 4:**
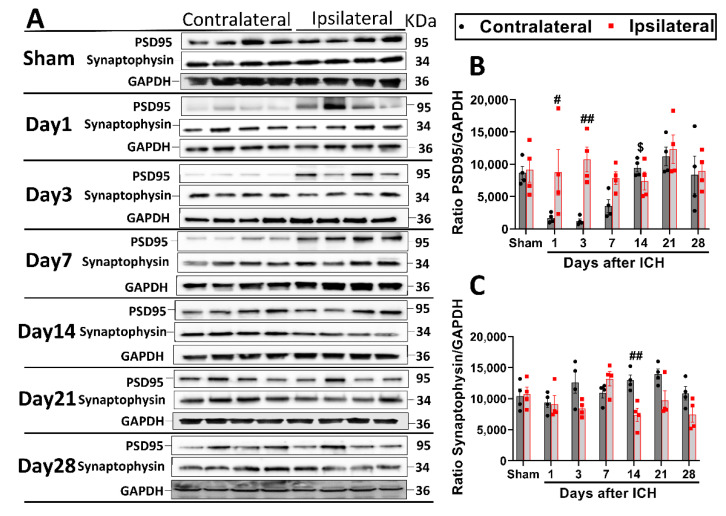
Synaptic dysfunction following ICH. Contralateral and ipsilateral regions isolated from different cohorts of mice were used for Western blotting to assess proteins for synaptic dysfunction. (**A**) Representative blots from different cohorts with different antibodies. A total of 25 µg of protein was loaded into each gel lane. GAPDH as a loading control, PSD95 as a marker for post-synaptic, and synaptophysin as a presynaptic marker. (**B**,**C**) Western blot analysis normalized to GAPDH; *n* = 4 mice per group. (**B**) PSD95 was significantly decreased on the contralateral side on days 1 and 3 compared to the ipsilateral side, suggesting an off-target effect to compensate for the loss of post-synaptic expression on the ipsilateral side. (**C**) A decreased trend was observed in synaptophysin from day 1 up to day 28 in the ICH group. On day 14, a significant decrease on the ipsilateral side was observed compared to the contralateral side. Two-way ANOVA followed by Sidak’s post hoc comparisons was used for analysis. Data are expressed as mean ± SEM, where *p* < 0.05 was considered significant. $ Difference within ICH groups vs. ICH on day 3 in the ipsilateral region ($ *p* < 0.05) and # Difference of contralateral and ipsilateral regions in each different time-points after ICH (# *p* < 0.05; ## *p* < 0.01).

PSD-95 did not show changes at the various time points after ICH compared with the sham group, but a significant decrease in the contralateral region compared with the ipsilateral side was observed on days 1 and 3 (Figure 4A,B). Synaptophysin, a presynaptic marker, showed a significant reduction on day 14 in the ipsilateral region compared with the contralateral side. Then, a decrease was observed until day 28, suggesting cofilin overactivation and formation of rods/aggregates might have resulted in synaptic loss and PSCI (Figure 4A,C). Nevertheless, synaptophysin showed an increase on the contralateral side up to day 21 and was significantly increased on day 14 compared to the ipsilateral side, indicating protective mechanisms to compensate for the loss of synapses around the hemorrhage or the loss of motor function. The results show that synaptophysin and PSD95 regulation changes might have contributed to the motor and cognitive decline after ICH.

### 3.5. Cofilin–Actin Rods/Aggregates and Microglial Activation in Mice after ICH

**Figure 5:**
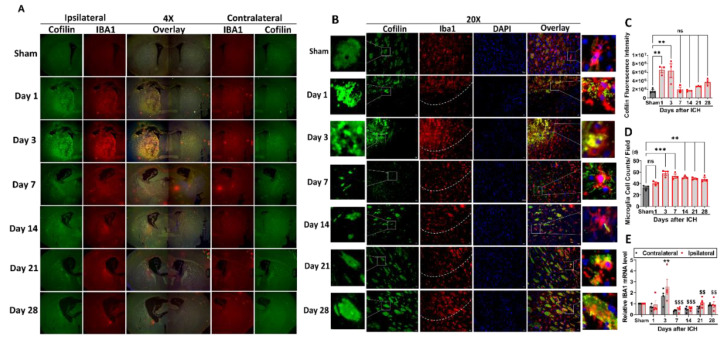
Cofilin and microglia activation in the perihematomal area. Brains from different cohorts of mice were fixed with paraffin for immunohistochemistry staining of cofilin and microglia. Another set of brains from different cohorts was used for mRNA analysis (**A**,**B**). Anti-cofilin (a marker of active cofilin) and anti-IBA1 (a marker of active microglia/macrophage) immunofluorescence microscopy analysis of paraffin-embedded brain sections (**A**). Scale bar of 200 µm and (**B**) Scale bar of 50 µm. (**C**,**D**) Quantification analysis of cofilin intensity and microglia counts were conducted using ImageJ software. (**E**) mRNA levels of IBA1 (microglia/macrophages) after ICH at different time points in the contralateral and ipsilateral areas. (**A**–**C**) Cofilin was significantly activated on days 1 and 3 after ICH compared with the sham group and decreased from day 7 to day 28. Cofilin–actin rods started to develop from day 7 to day 28. Intracellular cofilin rods/aggregates localized to activated microglia in the ICH groups and were associated with microglia morphological changes compared with the sham group. (**A**,**B**,**D**) Activated microglia illustrated by staining of IBA1 significantly increased from day 3 to day 28 after ICH compared with the sham group. In the sham group, microglia labeled with IBA1 ranged from ramified (inactive microglia) to de-ramify (pre-active microglia). In contrast, amoeboid microglia were mostly found around the hemorrhagic area in the ICH groups. (**E**) Microglia were significantly increased on day 3 after ICH. Expression of active microglia decreased from day 7 to day 28 compared with the sham group. Data are expressed as mean ± SEM, where *p* < 0.05 was considered significant; *n* = 3 mice per group. * Difference within groups relative to sham group (ns = not significant; ** *p* < 0.01; *** *p* < 0.001). $ Difference within ICH groups vs. ICH on day 3 in the ipsilateral region ($$ *p* < 0.01; $$$ *p* < 0.001). One-way ANOVA or two-way ANOVA followed by Sidak’s post hoc comparisons were used for analysis.

To assess the expression of cofilin rods/aggregates and microglial activation, coronal sections of paraffin-embedded mouse brain tissues were stained with anti-cofilin and anti-IBA1. Cofilin intensity/immunofluorescence was significantly upregulated on days 1 and 3 after ICH (*p* < 0.01; Figure 5A–C) compared with the sham group and decreased from day 7 to 28, which coincides with the protein expression increase from day 1 to day 3 and reduces from day 7 to day 28 in the ipsilateral side compared with the sham group. Cofilin–actin rods/aggregates started to develop from day 1 through day 28 (Figure 5A,B) and (Figure S3), suggesting the buildup of cofilin aggregates on days 1 and 3 and rods on days 7 to 28 (chronic period of ICH). Intracellular cofilin rods/aggregates were localized to the activated microglia in the ICH groups and were associated with microglia morphological changes compared with the sham group (Figure 5B). The number of activated microglia was significantly increased from day 3 (*p* < 0.001; Figure 5B) through day 14 (*p* < 0.01; Figure 5D) compared with the sham group, suggesting widespread neuroinflammation. Microglia labeled with IBA1 ranged from ramified (inactive microglia) to de-ramified (pre-active microglia) in the sham group. In contrast, amoeboid microglia were highly observed around the hemorrhagic area in the ICH groups, indicating the microglial active state (Figure 5B). mRNA levels of IBA1 significantly peaked on day 3 and gradually declined until day 28 in the ipsilateral region compared with the sham group. We observed no significant changes in IBA1 levels on the contralateral side from day 1 to day 28 (Figure 5E).

### 3.6. Inflammatory and Anti-Inflammatory Cytokines in Mice after ICH

**Figure 6:**
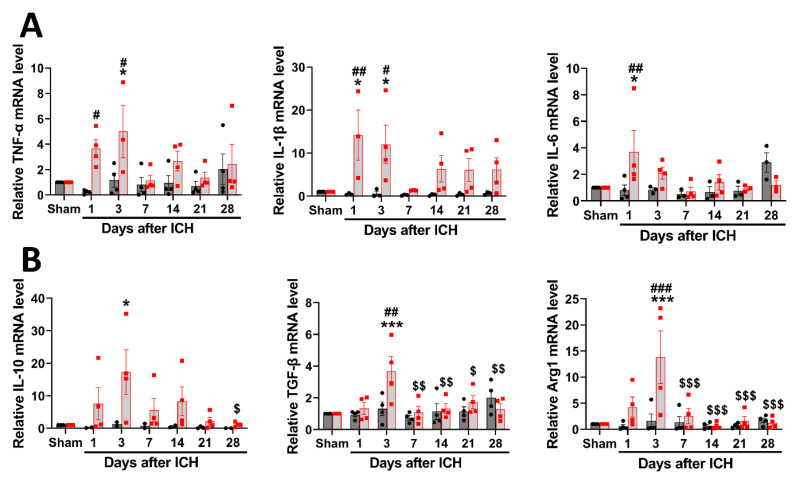
Inflammatory and anti-inflammatory mRNA levels. mRNA expression levels were detected in sham and ICH groups at various time points (1, 3, 7, 14, 21, and 28 days) by quantitative polymerase chain reaction in the ipsilateral and contralateral brain regions. (**A**) Inflammatory markers, e.g., TNF-α, IL-1β, and IL-6. (**B**) Anti-inflammatory markers, e.g., IL-10, TGF-β, and Arg1. The 18-S was used as a loading control. In the ipsilateral region (around the hemorrhage), (**A**) TNF-α was significantly increased on day 3, IL-1β on day 1 and day 3, and IL-6 on day 1 after ICH in the ipsilateral region. Expressions of TNF-α, IL-1β, and IL-6 decreased from day 7 to day 28 compared with the sham group. (**B**) IL-10, TGF-β, and Arg1 were significantly increased on day 3 after ICH in the ipsilateral region. IL-10, TGF-β, and Arg1 expressions decreased from day 7 to day 28 compared with the sham group. Two-way ANOVA followed by Sidak’s post hoc comparisons was used for analysis. Data are expressed as mean ± SEM, where *p* < 0.05 was considered significant. * Difference of sham vs. different time-points of ICH in the ipsilateral region (* *p* < 0.05; *** *p* < 0.001), $ Difference within ICH groups vs. ICH on day 3 in the ipsilateral region ($ *p* < 0.05; $$ *p* < 0.01; $$$ *p* < 0.001) and # Difference of contralateral and ipsilateral regions in each different time-points after ICH (# *p* < 0.05; ## *p* < 0.01; ### *p* < 0.001), (*n* = 4 mice).

Next, we examined mRNA levels for cytokines corresponding to M1/M2 microglial polarization. TNFα, IL-1β, and IL-6, as M1 markers, were measured in the sham and ICH groups at various time points (days 1, 3, 7, 14, 21, and 28) by quantitative polymerase chain reaction using 18-S as a loading control. In the ipsilateral region (around the hemorrhage), TNF-α was significantly increased on day 3, IL-1β on days 1 and 3, and IL-6 on day 1 after ICH compared to the sham group. The expressions of TNF-α, IL-1β, and IL-6 decreased from day 7 until day 28 compared with the sham group. In the contralateral region, no significant changes were observed (Figure 6A). We observed a significant increase in IL-10, TGF-β, and Arg1 (M2 markers) on day 3, and the levels reduced from day 7 until day 28 on the hematoma side, and no significant changes were detected on the other side (Figure 6B). It can be assumed that the augmented levels of both microglia phenotypes by day 3 are potentially contributing to the motor and PSCI after ICH.

### 3.7. Effect of ICH on the Blood Profile

**Figure 7:**
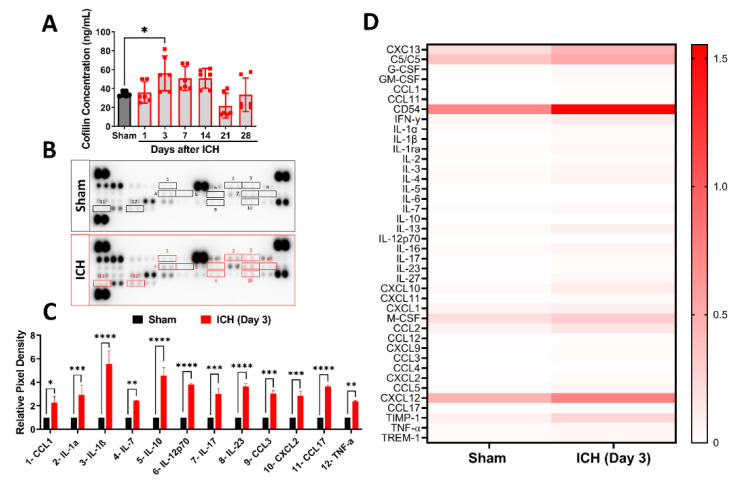
Blood cofilin levels and chemokine profile following ICH. Blood was collected from different cohorts of mice before sacrifice, and plasma cofilin by ELISA and serum cytokine proteome profiler arrays were performed. (**A**) Plasma levels of cofilin were significantly increased on day 3 compared with the sham group, and a gradual decrease was observed from day 7 to day 28 (*n* = 3 mice per group). (**B**) Representative bands of the proteome profiler mouse cytokine array in sham and ICH day 3 groups. (**C**,**D**) Quantitative analyses of detected cytokines and chemokines by proteome array; relative densities were normalized against the sham group (*n* = 2 membrane per group pooled from 4 mice per group). Heat map representing global variations in cytokine/chemokine responses in sham and ICH day 3, resulting in a significant increase in 13 cytokines/chemokines in the ICH group. Data are expressed as mean ± SEM, where *p* < 0.05 was considered significant. *Difference within groups relative to sham group (ns = not significant; * *p* < 0.05; ** *p* < 0.01; *** *p* < 0.001; **** *p* < 0.001). (One-way ANOVA followed by Dunnett’s *post hoc* comparisons or two-way ANOVA followed by Sidak’s post hoc comparisons).

To determine plasma cofilin levels after ICH, we collected mouse plasma at different time points (1, 3, 7, 14, 21, and 28 days) and compared it to the sham group. After ICH, plasma cofilin levels were significantly increased on day 3 (*p* < 0.0296, Figure 7A) compared to the sham group. Cofilin levels slightly decreased from day 7 until day 28 after ICH compared to day 3 and reached the same level as the sham group on day 28. These results indicate that ICH markedly increases cofilin levels in plasma, especially on day 3, implying peripheral inflammation. As cofilin peaked on day 3 after ICH, we further selected this time to analyze serum cytokines using a cytokine/chemokine array. The results show that 13 cytokines/chemokines were significantly increased in the ICH group on day 3 compared to the sham group (*p* < 0.001, Figure 7B–D). The pattern of increase in plasma cofilin with serum cytokines/chemokines may reflect a progressive pathological state, contributing to peripheral inflammation after ICH.

### 3.8. Histopathological, Hematoma, and Ventricular Volume Changes after ICH

**Figure 8:**
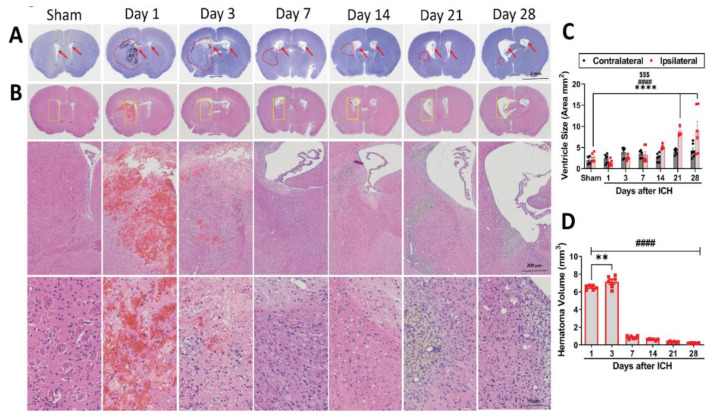
Changes in hematoma and ventricle size after ICH. Coronal sections were prepared from paraffin-embedded brains of sham, and ICH groups were stained for (**A**) Luxol fast blue, Cresyl violet (CV staining), and (**B**) Hematoxylin and eosin (H&E staining). Images were collected on the Olympus OlyVIA microscope and were displayed with a scale bar of 2 mm, 200 μm, and 50 μm. (**A**–**B**) Changes in ventricle sizes after ICH in contralateral and ipsilateral regions. (**B**) H&E staining showed histopathological changes on the ipsilateral side of the brain after ICH. (**C**) Increase in ventricle extension on days 21 and 28 compared with the sham group. (**D**) Hematoma volume significantly increased on day 3 and decreased on day 7 onwards. Data are expressed as mean ± SEM, where *p* < 0.05 was considered significant. * Difference of sham vs. different time-points of ICH in the ipsilateral region (** *p* < 0.01; **** *p* < 0.0001), $ Difference within ICH groups vs. ICH on day 3 in the ipsilateral region ($$$ *p* < 0.001) and # Difference of contralateral and ipsilateral regions in each different time-points after ICH (#### *p* < 0.0001), (two-way ANOVA followed by Sidak’s post hoc comparisons or one-way ANOVA followed by Dunnett’s post hoc comparisons ), (*n* = 3 mice).

Hematoma lesion volume and ventricle size were measured using Luxol fast blue/cresyl violet staining of sham and ICH brain sections. Cresyl violet staining showed clearance of hematoma but the concomitant enlargement of the ventricles from day 7 to day 28 after ICH in the hemorrhage side. Interestingly, this difference was also apparent on the contralateral side; we observed a gradual increase in ventricle size (Figure 8A,B,D). H&E staining showed histopathological changes in the hemorrhagic area. The total volume of ipsilateral ventricles, a sign of cerebral atrophy, was significantly larger in ICH mice on days 21 and 28 compared to sham (Figure 8C). The image of the sham group showed that neurons had clear spherical nuclei with abundant cytoplasm. On the hemorrhage side, the staining was light, with loose tissue and damaged nuclei from day 1 to day 28. The number of cells decreased and were swollen with structurally deformed cytoplasm. We observed extensive vacuolization on day 28 around the hemorrhagic area, consistent with cell damage compared with the sham group (Figure 8B). The results indicate that ventricular enlargement possibly occurs due to demyelination and causes brain atrophy and, eventually, chronic cognitive impairment after ICH. 

## 4. Discussion

In the present study, we investigated the cofilin expression in human ICH autopsy brains and the spatiotemporal signaling of cofilin following ICH in mice. We report for the first-time alterations in cofilin expression and cofilin localization within microglia in brain tissue of human ICH and mouse ICH longitudinal study stretching from day 1 to day 28. We showed that ICH causes motor deficits following ICH, and PSCI is observed in both acute and delayed phases. Furthermore, we demonstrate increased infarct volume from days 1 to 3 and ventricular enlargement after days 21 to 28. We demonstrate that cofilin overactivation on days 1 to 3 correlates with the SSH1 increase, followed by microglia activation on days 1 to 7. We also observed that brain mRNA levels of inflammatory and anti-inflammatory markers increased during the acute phase and decreased in the chronic phase. Furthermore, we observed blood cofilin levels increase on day 3, matching the increase in chemokine levels. The study outcomes suggest that cofilin overactivation due to SSH1 increase might play a role in microglial activation, neuroinflammation, and subsequent PSCI.

The cellular mechanisms that trigger pathological changes around hematomas in ICH patients are poorly understood. Multiple inflammatory cascades and cytotoxic mediators are involved [39]. These are known to cause neuronal cell death, microglial activation, and functional impairment, which are the features of secondary brain injury following ICH [40,41]. Understanding the inflammatory processes after ICH in humans may contribute to new therapeutic opportunities. Microglia are the resident immune cells of the brain that maintain brain homeostasis and regulate neuronal networks and synaptic plasticity. Microglia clear and engulf damaged debris through their phagocytic activity, but excessive microglial activation after brain injury is associated with pathological injury and neuroinflammation. The effects of toxic or protective microglia depend on their phenotype and the severity of the brain ictus [42,43]. One study has shown activated microglia in human brain sections from patients with spontaneous ICH, from day 1 of ICH to day 12, which was associated with alterations of microglial size and morphology [44]. Similarly, our study on humans and mice demonstrates that cofilin is overexpressed and colocalized with activated microglia around hematomas in the ICH group, with morphological changes contributing to neuroinflammation. In addition, studies have shown that activated cofilin is redistributed in the cytoplasm, leading to changes in the cell shape [45,46]. The knockdown of cofilin is associated with an increased amount of F-actin and a change in the microglial cell morphology [47]. These observations concord with our immunohistochemical results showing cofilin activation and redistribution and altered microglial morphology after ICH.

Moreover, the critical findings are the surge in inflammatory cytokines following ICH on day 3, impairing the M-2 anti-inflammatory phenotype and reducing repair mechanisms. In addition, M1 pro-inflammatory cytokines can aggravate brain injury and damage neurons and oligodendrocytes. Furthermore, microglia were observed to recover from their ameboid to ramified shapes on days 14, 21, and 28 on the ipsilateral side but were accompanied by the formation of cofilin rods localized in their processes (Figure 4). These results are consistent with the idea that cofilin rods/aggregates contribute to ICH, mainly by morphological alterations of microglia and neuroinflammation. The surge of inflammatory mRNA levels (TNF-α, IL-1β, and IL-6) within 1 day of ICH and lasting for 3 days provides evidence that cofilin overactivation is a starting point for widespread neuroinflammation. The decrease in cofilin expression beyond day 3 coincides with the decline in inflammatory markers. Nevertheless, the initial increase in anti-inflammatory markers (IL-10, TGF-β, and Arg1) on day 3 and then the reduction from day 7 to 28 depicts that the endogenous anti-inflammatory system does not recover from the initial onslaught of inflammation, hampering the recovery following ICH. It also implies that the inflammatory response in the CNS is a complex process that includes multiple steps with different mediators playing several roles at different times. It could be inferred that the immune response initially mounts an inflammatory response to clear damaged tissue but then tandemly switches to an anti-inflammatory response to promote tissue healing and recovery, but the severity of the injury overwhelms the antioxidant response. Plasma levels of cofilin increase, and plasma chemokines/cytokines surge within 3 days of ICH also correlates with cofilin brain levels. All these pieces of evidence point to cofilin overaction initiating inflammatory crises. Our published study where cofilin knockdown by siRNA before ICH reduced neuronal apoptosis, microglial activation, and oxidative stress and improved neurobehavioral deficits adds credence to our findings [34].

Furthermore, the idea that cofilin is associated with and is the center stage for neuroinflammation is further evidenced by the upstream and downstream signaling pathways found to be increased or decreased in our studies. To this end, SSHs is the leading player that activates cofilin. Three mammalian SSHs have phosphatase activity on cofilin. However, SSH1 is highly effective in activating the cofilin [23]. SSH1 has an N-terminal domain that interacts with cofilin, and Cys-393 residue is essential in eliminating the phosphate group from Ser-3 of the cofilin [48]. In addition, SSH1 can dephosphorylate LIMK1 and mitigate the LIMK1 enzymatic activity toward cofilin. SSH1 also has independent bundling activity to stabilize F-actin and stimulate the F-actin disassembly [49,50]. Singla et al. suggested that macrophage growth factor initiation could activate cofilin and control actin dynamics by the SSH1 pathway [51]. LIM-kinase is an actin-binding protein that phosphorylates cofilin at Ser-3, reducing actin-binding and depolymerizing cofilin activities [52,53]. LIMK1/LIMK2 regulates cofilin phosphorylation activity; LIMK1 phosphorylates cofilin by ras-linked C3 botulinum substrate 1 (Rac1), while LIMK2 inactivates cofilin through Rho and cell division cycle 42 (CDC42) [54]. Downregulation of LIMK1 restrains the lamellipodium development produced by Rac1 or insulin. Therefore, LIMK1 and LIMK2 phosphorylate cofilin via the Rac1 and Rho/CDC42 pathways [55]. An increase in SSH1 and a decrease in LIMK1 and pLIMK are conducive to cofilin activation and could be a sequel for neuroinflammation.

The occurrence of PSCI after ICH suggests diverse pathophysiologic mechanisms triggering this phenomenon. Our study shows that PSCI occurs in mice in the acute and chronic phases after ICH, with findings in parallel with those observed in human ICH patients [56]. In addition, we noted that mice suffer from severe PSCI in the acute and chronic phases after ICH. The observed increase in infarct volume from day 1 to 3, which was taken over by ventricular loss after day 21 to 28, suggests that PSCI is the possible outcome of neuronal loss and tissue damage. The signaling pattern of decreased synaptophysin on day 14 in the ipsilateral region and PSD95 in the contralateral area indicates PSCI following ICH. Since cofilin overactivation or rods/aggregates lead to a reduction in synaptophysin and PSD95, it might be the possible cause of synaptic dysfunction and PSCI observed in our results. The results are in line with other studies, in which cognitive impairment occurs in the acute (5 days) [57] or chronic period (28 days [58] or 35 days [18]) after ICH. A previous study suggested that cofilin rod formation impairs synaptic structure and function in both in vivo and in vitro ischemic models [26]. Won et al. showed that cofilin activation leads to cofilin–actin rods in neuronal processes in four different ischemic mouse models implicating oxidative stress main culprit due to cofilin–actin rod formations [30]. A recent study showed that cofilin rod formation is significantly increased in the cortical core and penumbra after transient middle cerebral artery occlusion, causing degradation of microtubule-associated protein-2 (MAP2) and apoptosis [59]. Inhibiting cofilin rod aggregation by overexpression of LIMK rescued the MAP2 and attenuated the apoptosis, suggesting cofilin in direct inhibition as a treatment option for the ischemic stroke [59]. Our findings are in consonance with the aforementioned studies, suggesting that cofilin rods/aggregates might have been associated with microglial activation and neuroinflammation, subsequently impacting synaptic plasticity and PSCI. 

Dysregulation of cofilin signaling or expression has been implicated in several neurological conditions, including ICH [33,34], ischemic stroke [60], and traumatic brain injury (TBI) [61]. Given the similarities between ICH, ischemic stroke, and TBI in terms of the initial injury and subsequent inflammatory response, it is likely that cofilin dysregulation or overexpression may play a role in the pathophysiology of neural injury in these conditions. Cofilin overexpression has been linked to several key features of ischemic stroke pathophysiology, including excitotoxicity, oxidative stress, and inflammation. Cofilin rod aggregation in ischemic stroke can contribute to excitotoxicity by promoting dendritic spine loss and disrupting the cytoskeleton, impairing synaptic plasticity and neuronal death [26]. Cofilin can also interact with other signaling pathways that enhance oxidative stress and inflammation, contributing to the neurodegeneration [55]. Similarly, in TBI, the injury can cause a complex cascade of events that result in tissue damage and cell death [62]. Cofilin overexpression may contribute to this damage by promoting inflammation and disrupting cellular signaling pathways, which can aggravate tissue injury and contribute to secondary damage. Therefore, the results of cofilin signaling in ICH might help understand the pathophysiology of neural injury in ischemic stroke and TBI.

Several limitations need to be mentioned. First, sex and age are essential features that impact the ICH outcomes. Studies have reported that women with spontaneous ICH show less brain edema than men [63]. Female mice with ICH also display lesser neurological deficits and brain edema than male mice, suggesting sex differences in ICH, likely mediated by estrogen-reduced iron and hemoglobin-induced neurotoxicity [64,65,66]. Aged rats with ICH show more severe neurological deficits and brain swelling than young rats, indicating that age has a crucial effect on response to ICH [67]. Therefore, future studies involving aged mice are warranted to study their impact on cofilin signaling after ICH. Moreover, later time points, such as 3 months of survival after ICH, should also be considered to evaluate long-term pathological alterations, particularly cognitive deficits. We noted that cofilin was also overexpressed in the neuropil area, a finding that merits further studies on cofilin’s impact on neuronal, astrocytic, and oligodendrocyte functions. Finally, there is a limitation of not showing the direct role of cofilin by either blocking the phenotype or knocking out the gene. Such a study would have conclusively established the direct role of cofilin in neuroinflammation and other outcomes. 

In conclusion, the results of the present study show for the first time that hemorrhagic brain injury-induced cofilin rods/aggregates are associated with microglial activation and microglial morphological alterations in humans and mice. These results support the idea that longitudinal cofilin signaling is at the center stage of microglial activation, neuroinflammation, motor impairments, and PSCI, offering novel potential therapeutic avenues for ICH.

## Data Availability

All data needed to evaluate the conclusions in the paper are present in the paper and/or the Supplementary Materials.

## Supplementary Materials

The following supporting information can be downloaded at: https://www.mdpi.com/article/10.3390/cells12081153/s1, Table S1. Cases used for cofilin and microglial immunostaining. Based on the final diagnosis, the cases were classified as control or hemorrhagic brain injury. Table S2. Shows primer sequence for each gene. Figure S1. Cofilin and microglial counts in male and female ICH patients. Immunofluorescence analysis of cofilin intensity and microglia counts in male and female ICH patients compared to the control group were performed using ImageJ software. Data are expressed as mean ± SEM, where *p* < 0.05 was considered significant (*n* = 8 control, *n* = 10 ICH, unpaired Student’s *t*-test). * *p* < 0.05, *** *p* < 0.001, **** *p* < 0.0001). Figure S2. Cofilin localization in and around the nucleus of human ICH patient brain sections (Scale bar 20 µm). Immunohistochemistry staining indicated that cofilin localized around and in the nucleus of ICH patients. Figure S3. Immunohistochemistry staining of cofilin and F-actin around the hemorrhagic region after ICH. Anti-cofilin (a marker of active cofilin) and anti-F-actin (a filamentous actin) were used to stain the paraffin-embedded brain sections (Scale bar 200 µm). The data showed cofilin-actin rods/aggregates after ICH at different time points (sham, days 1, 3, 7, 14, 21, and 28). References [68,69,70,71,72,73,74] are referred to in Supplementary Materials.
