# Supplementary material for: Spatiotemporal Cofilin Signaling, Microglial Activation, Neuroinflammation, and Cognitive Impairment Following Hemorrhagic Brain Injury
Source: Cells. 2023 Apr 13;12(8):1153. doi: 10.3390/cells12081153 (PMC10137307; doi:10.3390/cells12081153)
Supplement: Supplementary file 1 [file cells-12-01153-s001.zip › cells-2318342-supplementary.pdf]

cells MDPI

Supplementary Material

Spatiotemporal Cofilin Signaling, Microglial Activation, Neuroinflammation and Cognitive Impairment Following Hemorrhagic Brain Injury

Daniyah A. Almarghalani [1], Xiaojin Sha[2], Robert E. Mrak[3] and Zahoor A. Shah[4,*]

1 Department of Pharmacology and Experimental Therapeutics College of Pharmacy and Pharmaceutical Sciences, University of Toledo, Toledo, 43614, OH, USA
2 Department of Pathology, College of Medicine, The University of Toledo, Toledo, 43614, OH, USA
3 Department of Laboratory Medicine and Pathology, University of Washington, Seattle, 98195, WA, USA
4 Department of Medicinal and Biological Chemistry, University of Toledo, Toledo, 43614, OH, USA
* Correspondence: zahoor.shah@utoledo.edu

Table S1. Cases used for cofilin and microglial immunostaining. Based on the final diagnosis, the cases were classified as control or hemorrhagic brain injury.

Case.	Classification	Age (y)	Gender	Postmortem Interval (h)	Hemorrhage Brain Area
R1	Hemorrhage	68	M	48	massive left-sided cerebral, right-sided cerebellar
R2	Hemorrhage	57	M	15	massive right intracerebral
R3	Hemorrhage	48	F	15	left cerebral hemorrhage subarachnoid hemorrhage (left cerebral hemisphere, cerebellum)
R4	Hemorrhage	58	M	68	Duret hemorrhage of pons, right parietal and temporal lobe hemorrhage
R5	Hemorrhage	63	M	24	intracerebral hemorrhage, bilateral
R6	Infarction, Hemorrhage	38	M	24	Duret hemorrhage, pons subgaleal hemorrhage, left side
R7	ICH	68	F	48	subarachnoid hemorrhage
R8	ICH	49	F	24	left thalamic hemorrhage
R9	ICH	65	F	24	intraparenchymal pontine hemorrhage with extravasation to the subarachnoid space
R10	Recurrent ICH	71	M	24	recurrent ICH, ICH- right front lobe, left basal ganglia
RN1	Control	43	M	24	myocardial infarction, ARDS
RN2	Control	58	F	66	ruptured abdominal aortic aneurysm
RN3	Control	83	M	48	respiratory failure, heart failure angiodysplasia, diverticulosis, acute hemorrhagic gastritis acute tubular necrosis, chronic pyelonephritis
RN4	Control	65	M	48	pulmonary embolism
RN5	Control	58	F	72	hepatic cirrhosis with portal hypertension ruptured esophageal varices
RN6	Control	43	F	24	Bilateral pulmonary artery thromboemboli
RN7	Control	24	F	72	acute heart and respiratory failure
RN8	Control	55	M	24	multiple pulmonary artery thromboemboli sickle cell disease

Table S2. Shows primer sequence for each gene.

Targets	Primers	Sequences	References
18S	Forward (Sense)	5′-CCTGGATACCGCAGCTAGGA-3′	[68]
	Reverse (Antisense)	5′-GCGGCGCAATACGAATGCCCC-3′	
IL-1β	Forward (Sense)	5′-CCAGCTTCAAATCTCACAGCAG-3′	[69]
	Reverse (Antisense)	5′-CTTCTTTGGGTATTGCTTGGGATC-3′	
TNF-α	Forward (Sense)	5′-CACAGAAAGCATGATCCGCGACGT-3′	[69]
	Reverse (Antisense)	5′-CGGCAGAGAGGAGGTTGACTTTCT-3′	
IL-6	Forward (Sense)	5′-TCCAGTTGCCTTCTTGGGAC-3′	[69]
	Reverse (Antisense)	5′-GTACTCCAGAAGACCAGAGG-3′	
IL-10	Forward (Sense)	5′-TGGCCCAGAAATCAAGGAGC-3′	[69]
	Reverse (Antisense)	5′-CAGCAGACTCAATACACACT-3′	
TGF-β	Forward (Sense)	5′-GACCGCAACAACGCCATCTA-3′	[69]
	Reverse (Antisense)	5′-GGCGTATCAGTGGGGGTCAG-3′	
Arg1	Forward (Sense)	5′-CCACGGTCTGTGGGGAAAGCCAAT-3′	[70
	Reverse (Antisense)	5′-CTGCCAGACTGTGGTCTCCACCCA-3′	
IBA1	Forward (Sense)	5′-GTCCTTGAAGCGAATGCTGG-3′	[71]
	Reverse (Antisense)	5′-CATTCTCAAGATGGCAGATC-3′	

Neurobehavioral tests:

A- Neurological deficit scoring (NDS)

NDS is a 28-point scoring system used to evaluate sensory and motor deficits following ICH [72]. Seven tests were graded from zero (no deficit) to four (severe deficit) and evaluated after ICH, seven tests: body symmetry, gait, climbing, circling behavior, whisker response, compulsory circling, and front limb symmetry

A- Rotarod test

Rotarod was used to evaluate the deficits in motor coordination following ICH as previously described [34]. In brief, a mouse was placed on a moving rod of rotarod that was adjusted to rotate at 1 RPM, and speed increased by 1 RPM every 10 s until the mouse fell off. The trial ends when the mouse falls from the rod, and the time is recorded. Animals were trained on the Rotarod 3–7 days and 24 h before the surgery, considered the baseline and after ICH (each animal was tested three times per trial, and the average value was utilized for the statistical analysis).

A- Grip strength test

Grip strength was used to measure the strength of the forelimb muscles of the mouse following ICH. Animal forelimbs were placed on a pull bar assembly by holding it by the tail. The maximum force exerted by the animal and displayed on the digital screen was recorded (each animal was tested three times per trial, and the mean value was used for statistical analysis). The reported scores were recorded three days before ICH was used as the baseline and after ICH.

A- T-maze

T-maze was used to assess spatial learning and cognitive impairment [73]. We used the spontaneous alternation protocol described previously [74] with minor modifications. Each trial started by placing the mouse on the central arm and exploring both arms for 2 min; once the mouse chose a particular goal arm, the arm was blocked, and the mouse was removed after 30 s. The mouse was placed back into the cage for 30 s, and the block was removed from the maze. Then the mouse was placed in the start arm, allowing the mouse to choose the arm. We run two sets/day for four days. The correct choice was recorded when the second run was different from the first in a given set, and the incorrect choice was recorded when the mouse chose the same arm similar to the previous run. We calculated the percentage of alteration and the rate of side preference.

%Alteration= (Correct choice/Total set)*100
%Side Preference= (Preferred side/ Total run)*100

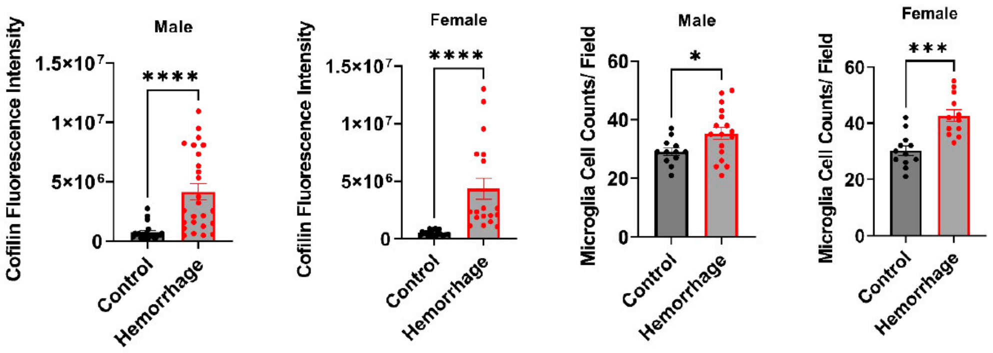

Figure S1. Cofilin and microglial counts in male and female ICH patients. Immunofluorescence analysis of cofilin intensity and microglia counts in male and female ICH patients compared to the control group were performed using ImageJ software. Data are expressed as mean ± SEM, where $p < 0.05$ was considered significant ($n = 8$ control, $n = 10$ ICH, unpaired Student's t-test). * $p < 0.05$, *** $p < 0.001$, **** $p < 0.0001$).

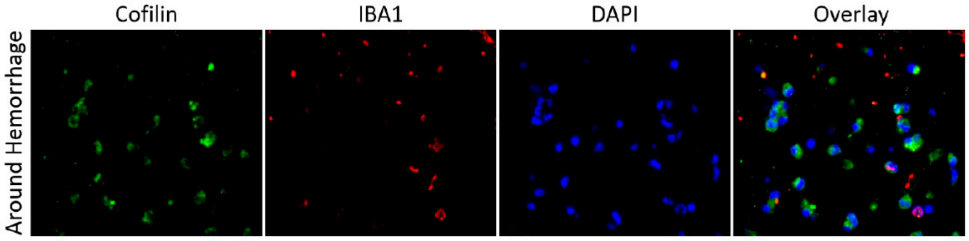

Figure S2. Cofilin localization in and around the nucleus of human ICH patient brain sections (Scale bar 20 µm). Immunohistochemistry staining indicated that cofilin localized around and in the nucleus of ICH patients.

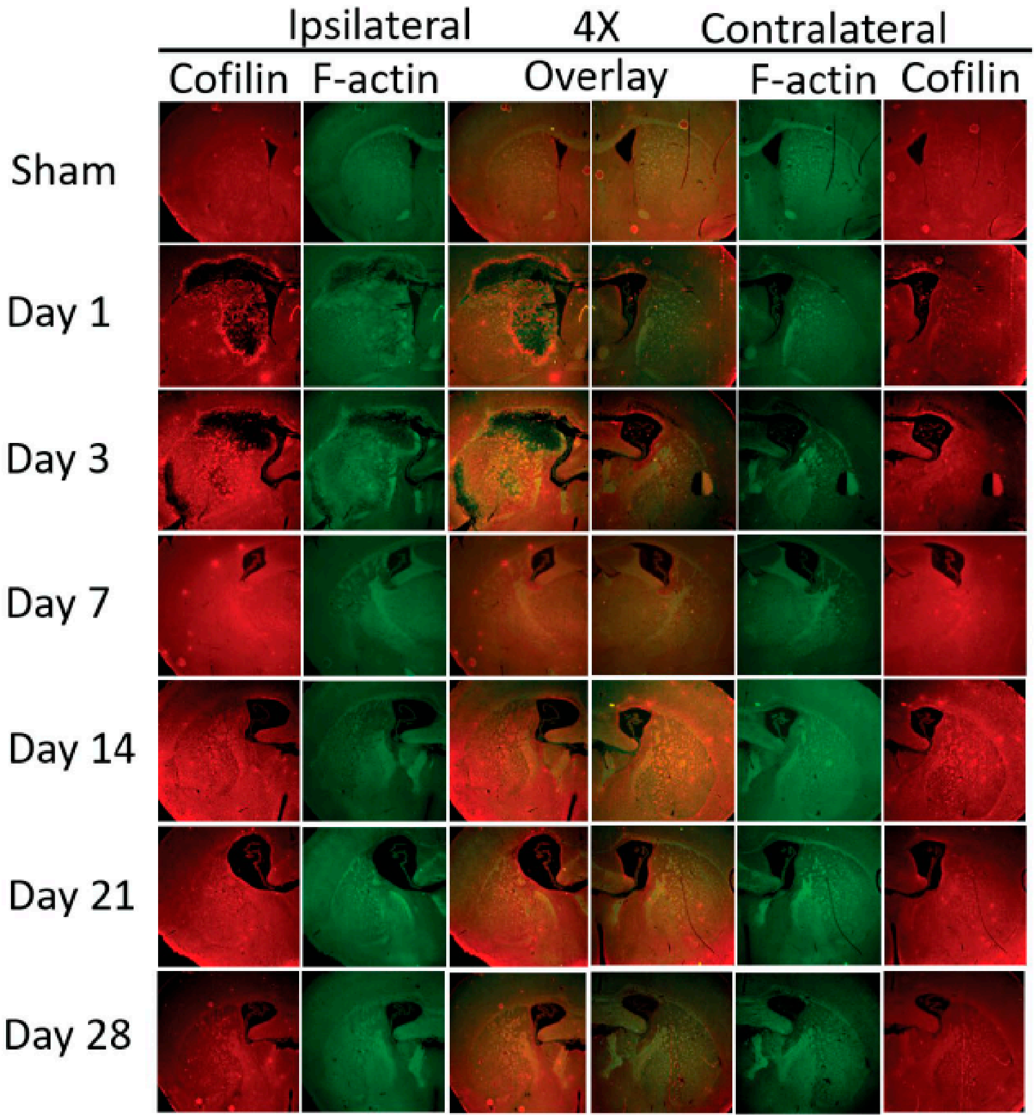

Figure S3. Immunohistochemistry staining of cofilin and F-actin around the hemorrhagic region after ICH. Anti-cofilin (a marker of active cofilin) and anti-F-actin (a filamentous actin) were used to stain the paraffin-embedded brain sections (Scale bar 200 μm). The data showed cofilin-actin rods/aggregates after ICH at different time points (sham, days 1, 3, 7, 14, 21, and 28).
